# Facial Pre-Touch Space Differentiates the Level of Openness Among Individuals

**DOI:** 10.1038/s41598-019-48481-x

**Published:** 2019-08-15

**Authors:** Soheil Keshmiri, Masahiro Shiomi, Kodai Shatani, Takashi Minato, Hiroshi Ishiguro

**Affiliations:** 1 Advanced Telecommunications Research Institute International (ATR), Kyoto, Japan; 2 Graduate School of Engineering Science, Osaka University, Osaka, Japan

**Keywords:** Human behaviour, Computational science

## Abstract

Social and cognitive psychology provide a rich map of our personality landscape. What appears to be unexplored is the correspondence between these findings and our behavioural responses during day-to-day life interaction. In this article, we utilize cluster analysis to show that the individuals’ facial pre-touch space can be divided into three well-defined subspaces and that within the first two immediate clusters around the face area such distance information significantly correlate with their openness in the five-factor model (FFM). In these two clusters, we also identify that the individuals’ facial pre-touch space can predict their level of openness that are further categorized into six distinct levels with a highly above chance accuracy. Our results suggest that such personality factors as openness are not only reflected in individuals’ behavioural responses but also these responses allow for a fine-grained categorization of individuals’ personality.

## Introduction

Personality, with its signatures already etched on our brain^1^, is what defines us as individuals and determines our responses to psychological stressors^2^. Recent findings on its traits^3^, types^4^, and neural correlates^5^ have substantially advanced our understanding about individuality^6^ that can be reliably identified across different languages and cultures^7^. For instance, the big-5 or five-factor-model (FFM)^8^ has been shown to provide a good predictor for such patterns of behaviour as well-being and mental health, job performance and marital relations^9^, as well as the clinical assessments of personality disorders^10^.

In this respect, there is ample evidence that point at the effect of personality on our social development^11,12^ and embodied interactions^13–15^ that is not affected by the nature of interacting agency^16^. These observations beg the question of whether personality also influences such behavioural responses as personal space^17^ and interpersonal distance^18,19^. The significance of such a scrutiny is clarified by considering the findings that emphasize the positive socioemtional effect of physical interaction on our wellbeing^20–24^.

However unlike the findings that identify the correspondence between body and such internal states as emotions^25–27^, lack of consensus on the interplay between personality and personal space^28,29^ does not warrant an informed conclusion on the influence of the personality traits on our behavioural responses.

In this article, we address this shortcoming through cluster analysis of the individuals’ facial pre-touch distance. We consider the facial area touch interaction as opposed to other body parts that are more openly shared during social interactions (e.g., shoulder patting) due to higher sensitivity of people around their face which makes the facial boundary to play a substantial role in understanding the people’s behavioural responses within the context of touch interaction. We show that the individuals’ facial pre-touch space can be divided into three well-defined subspaces. Within the first two immediate clusters around the face area, we identify that such distance information significantly correlate with individuals’ openness in FFM. We also show that the individuals’ facial pre-touch space can predict their level of openness that are further categorized into six distinct levels with a highly above chance accuracy. Our results suggest that such personality factors as openness are not only reflected in individuals’ behavioural responses but also these responses allow for a fine-grained categorization of individuals’ personality.

## Materials and Methods

### Participants

Fifty younger adults (M = 21.83, SD = 1.53) participated in our experiment. These individuals were paired into four distinct categories: female touchers and evaluators (FF), female touchers and male evaluators (FM), male touchers and female evaluators (MF), male touchers and male evaluators (MM). Data from three participants were not usable and therefore we excluded their corresponding two pairs from further analyses. This experiment was carried out with written informed consents from all subjects.

We recruited the participants through a local commercial recruiting website. Our participants were not limited to university students and came from different occupational background.

### Ethics statement

This study was carried out in accordance with the recommendations of the ethical committee of the Advanced Telecommunications Research Institute International (ATR) with written informed consent from all subjects in accordance with the Declaration of Helsinki. The protocol was approved by the ATR ethical committee (approval code:17-601-4).

### Paradigm

**Figure 1:**
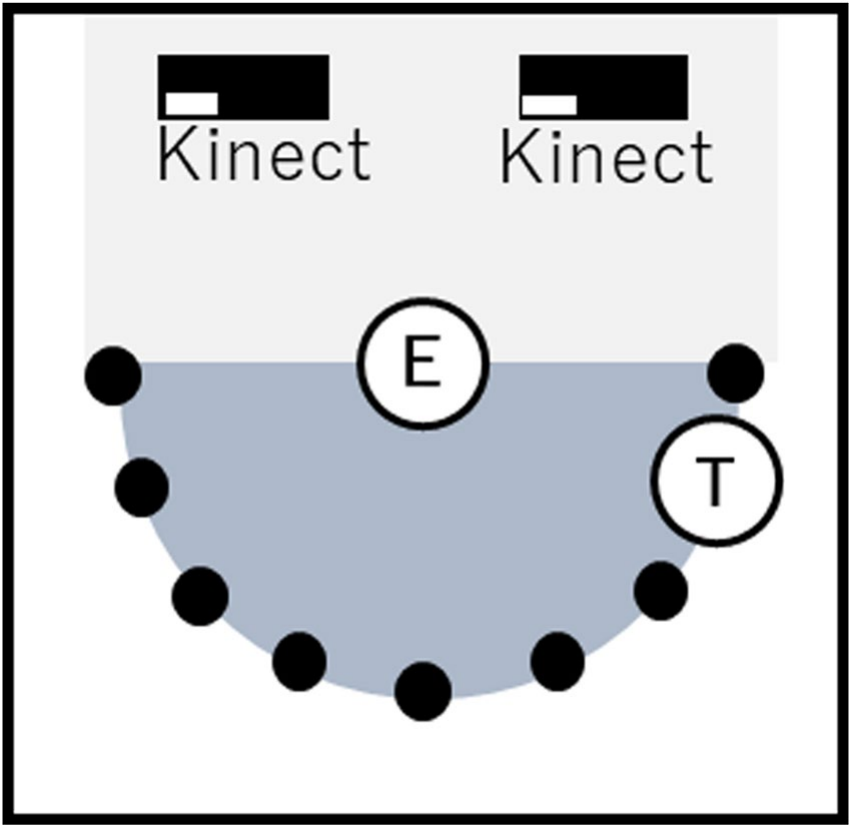
Predetermined toucher-evaluator interaction positions. In this setting, the toucher (i.e., T) moves along the positions 0 through 8 and stretches his hand toward the face of the evaluator (i.e., E) who is seated in the middle. The two Kinect V2 sensors mounted behind the evaluator collect the joint and the head positions of the toucher and the evaluator. The location of two Kinect V2 sensors that were mounted behind the evaluators’ seat to automatically track the touchers’ hand and the evaluator’s face positions are visible in this figure.

We conducted a facial pre-touch distance experiment to study whether individuals’ facial area pre-touch space can predict their personality traits in FFM. For this purpose, we acquired the facial pre-touch distances that were measured between the hand of a toucher and the face of a person who was about to be touched (evaluator). Figure 1 shows an instance of the experiment. The evaluator was seated on a chair in the middle of the experimental room and the toucher stood close to the evaluator in a distance that was adjusted based on the arm’s length of each of the touchers in our experiment. The nine approaching positions from which the toucher reached for the face-area of the evaluator are shown in this figure (positions 0 through 8). In our experimental setup, the touchers slowly stretched their hand toward the evaluators’ face. While doing so, they freely decided their initial hand position and their approaching angle. When the evaluators felt that the touchers’ hand were exceeding their comfort zone and wanted them to stop, they clicked a mouse bottom whose clicking sound was audible to the touchers. We instructed the touchers to immediately stop getting their hand any closer to the evaluators’ face once they heard the mouse clicking sound. We then measured the distance between the touchers’ hand and the evaluators’ face and used these measured distances as the minimum comfortable pre-touch distance of the individuals (i.e., their behavioural-based facial pre-touch boundary). We did not fix the number of pre-touch interactions and allowed the participants to continue as long as their allocated time permitted. Each pairs of toucher-evaluator participated in a two-hour trial during which one of them played the role of the evaluator for the first one-hour and the other was the toucher (i.e., approximately 6.67 minutes per touch-interaction spot in Fig. 1) and then switched their roles during the second one-hour period. While interacting, we asked the participants to look at the center of the approaching hand from their own perspective (i.e., palm of the hand for the evaluator and the back of the hand for the toucher) and to keep neutral facial expression and suppress reactions toward the touch during their interaction. The average number of trials per participants was M = 288.02 (SD = 78.02, CI = [265.11 310.93]).

### Data acquisition

We used two Kinect V2 sensors that were mounted behind the evaluators’ seat (Fig. 1) to track the touchers’ hand and the evaluators’ face positions. We collected the 3D positions of each joint of the touchers (including the center of their hands) and the 3D head position of the evaluators. We also recorded the timing of the evaluators’ mouse clicks that signalled the touchers to stop getting their hand any closer to the evaluators’ face. In order to calculate the evaluators’ facial pre-touch distances (in cm), we subtracted the size of the touchers’ hand (measured prior to the commencement of the experiment) from the average Japanese face size (i.e., 9.0 cm for female and 10.0 cm for males^30^).

Giancola *et al*.^31^ suggested that Kinect sensors are suitable for applications in which the joint position accuracy does not exceed a few cm. However in their study, they focused on the accuracy of a whole body tracking algorithm in a upper-limb rehabilitation scenario. Our experiment differed from their setting in which we considered the interaction space between the touchers’ hand and the evaluators’ face. Therefore, we employed (unlike Giancola *et al*.^31^) two Kinect sensors for data acquisition, thereby bypassing the use of markers on touchers’ hand and the evaluators’ face to prevent their potential confounding effect on participants’ pre-touch feelings. To increase the accuracy of the detected joint positions, we further calibrated the relative positions of these two sensors and used their absolute positions to integrate their joint positions data. In the event of one Kinect sensor’s failure, we used the other sensor if its estimates were continuous and stable.

To test for the instrument’s reliability, we used a Japanese version of the Ten-Item Personality Inventory (TIPI-J)^32^. Since the TIPI-J has only two items for each domain, the authors in^32^ used within-scale inter-item correlations for evaluating the internal consistency of each scale than the Cronbach’s alpha coefficients^33^. Therefore, we did not evaluate Cronbach’s alpha, but we believe that the validity of the TIPI-J is already evaluated via original authors.

### Analysis

We first utilized Kruskal-Wallis test to verify that there was no effect of four paired gender groups (i.e., FF, FM, MF, and MM) on participants’ facial pre-touch distances. Anther factor that needed further verification was the potential effect of the familiarity between the pairs of interacting participants. Specifically, it was important to determine whether the facial space between these individuals shrank as they interacted throughout their session. For this purpose, we used the averages of the first and the last 10 facial pre-touch distance measurements of each participants and applied Wilcoxon rank sum test on these two sets of average distances. We found that the effect of gender and the familiarity between interacting pairs were non-significant (for details, see supplementary material (SM)).

Our analysis of the potential correspondence between facial pre-touch distance and the FFM personality traits included three steps: (1) cluster analysis of the participants’ facial pre-touch distance to determine their potential spatial clusters around the face area (2) Spearman correlation between pre-touch distances of these clusters and the individuals’ FFM personality scores (3) classification of the individuals’ personality traits based on the results of the correlation analysis in step (2).

#### Cluster analysis of the facial pre-touch distances

To determine whether the individuals’ facial pre-touch distances had a potential spatial pattern around the face area, we applied cluster analysis on these pre-touch distances. To choose between parametric (e.g., gaussian mixture model (GMM)) and non-parametric (e.g., K-means algorithm) clusterings, we first applied the Lillifors test with Monte Carlo approximation to determine whether individuals’ facial pre-touch distances (both their actual as well as log-transformation) followed a normal distribution. The test rejected the presence of normality at 5.0% significance level. Therefore, we adapted non-parametric analyses in present study.

We used the K-means algorithm^34^ for cluster analysis of the participants’ facial area pre-touch distances. We applied this clustering step on the entire pre-touch distance data (i.e., all the participants combined). The basic principle underlying this algorithm is to group the data points into a specified number of clusters in such a way that the Euclidean distance between the members of these clusters to their corresponding cluster center is minimized. We used participants’ pre-touch distances (in cm) along with the azimuth and elevation angles (in degrees) associated with these distances as inputs to the K-means algorithm. In order to determine the number of clusters, we utilized Akaike and Bayesian information criteria (AIC and BIC) and checked for cluster number K = 1, …, 5. Both AIC and BIC indicated that K = 3 best suited our data. Therefore, we used this value for clustering the participants’ facial pre-touch distances.

#### Correlation analysis of the facial pre-touch distances and FFM scores

We used the resulting three clusters and performed Spearman correlation between pre-touch distances that were assigned to each of these clusters and their corresponding FFM scores (i.e., extraversion, agreeableness, conscientiousness, openness, and neuroticism) of the participants. Specifically, we first computed the average facial pre-touch distance of each individual in each cluster and then used these average distances along with the FFM scores that were within [1 … 7] real-valued intervals (e.g., openness = 3.78) for correlation analysis. We found that the participants’ openness scores and their pre-touch distances showed significant anti-correlation in the first two immediate clusters around the face area. To further verify the observed anti-correlations in these two clusters, we computed their 95.0% bootstrap (10,000 rounds) confidence intervals. For the bootstrap test, we considered the null hypothesis *H0: there is no correlation between the individuals’ facial pre-touch distances and their openness scores* and tested it against the alternative hypothesis *H1: there is a significant correlation between the individuals’ facial pre-touch distances and their openness scores*. We reported the mean, standard deviation, and the 95.0% confidence interval for these tests. We also computed the p-value of these tests as the fraction of the distribution that was more extreme than the actually observed anti-correlation values. For this purpose, we performed a two-tailed test in which we used the absolute values so that both the positive and the negative correlations were accounted for.

#### Classification of the individuals’ personality traits

Since we found that the participants’ openness scores and their pre-touch distances showed significant anti-correlation in the first two immediate clusters around the face area, we excluded the outermost cluster around the face area and primarily used the other two clusters (for correlation results associated with the third cluster as well as other FFM scores than the openness, see SM). Since we wanted to determine whether it was possible to determine the level of openness of an individual based on their measured facial pre-touch distances, we first grouped the participants into six openness levels based on their openness scores that were within [1 … 7] real-valued intervals (e.g., openness = 3.78). We calculated these groups using the following boundaries:

$$openness=\{\begin{array}{ll}\mathrm{1,} & {\rm{if}}\,score\,\le \,{\rm{2.0.}}\\ \mathrm{2,} & {\rm{if}}\,{\rm{2.0}}\, < \,score\,\le \,{\rm{3.0.}}\\ \mathrm{3,} & {\rm{if}}\,{\rm{3.0}}\, < \,score\,\le \,{\rm{4.0.}}\\ \mathrm{4,} & {\rm{if}}\,{\rm{4.0}}\, < \,score\,\le \,{\rm{5.0.}}\\ \mathrm{5,} & {\rm{if}}\,{\rm{5.0}}\, < \,score\,\le \,{\rm{6.0.}}\\ \mathrm{6,} & \mathrm{ifscore} > 6{\rm{.0.}}\end{array}$$

We then used these six groups and applied nine different classification methods to determine the utility of the participants’ pre-touch distance information in predicting their openness level in the first two immediate clusters around the face area. They were support vector classifier (SVC), quadratic discriminant analysis (QDA), adaboost, logistic regression (LR), naive Bayes (NB), random forest (RF), decision tree (DT), k-nearest-neighbour (KNN), and linear discriminant analysis (LDA). We used the participants’ pre-touch distance (in cm) along with their azimuth and elevation angles (in degrees) as input features to these algorithms. The preprocessing of the models’ input features included the scaling of these features (column-wise) within [0, …, 1] using $\frac{f-min({C}_{i})}{max({C}_{i})-min({C}_{i})}$ where *C*_*i*_, *i* = 1,…3, refers to the i^*th*^ column in the feature vector (i.e., *C*_1_, *C*_2_ for azimuth and elevation angles and *C*_3_ for the pre-touch distance and *f* identifies a specific feature value that is scaled. The output from these classifiers were their predicted openness level of the participants (i.e., levels 1 through 6). Given the six levels of openness, the chance level accuracy was ≈16.67%.

For comparison of the classifiers’ accuracy, we performed 200 simulation runs in which we randomly split the pre-touch distances (per cluster) to 70.0% train and 30.0% test sets. We also ensured that a balanced proportion of each of the six labels were split between these train and test sets. In each run, we used the same split of train and test sets and applied the above nine classifiers. We used the train set for training these classifiers and the test set to compute their prediction accuracy, precision, recall, and F1-score. We then used the 200 predictions by each of these algorithms and applied Friedman’s test that was followed by posthoc Wilcoxon signed rank to determine the classifier with the highest accuracy. Our results indicated that KNN significantly outperformed the other classifiers which we further verified it by computing the 99.0% bootstrap (10,000 rounds) confidence intervals of the accuracies of these models. For the bootstrap test, we considered the null hypothesis *H0: there is no difference between the average accuracy of KNN and the other models* and tested it against the alternative hypothesis *H1: KNN’s average accuracy is significantly higher than those of the other models*. We reported the mean, standard deviation, and the 99.0% confidence interval for these tests. Therefore, we adapted KNN for our main analysis (for details of this comparative analysis, see SM).

We used the KNN’s predictions during 200 simulation runs (per cluster) and applied Kruskal-Wallis test to determine whether the KNN accuracy was affected by different levels of participants’ openness. This was followed by the posthoc Wilcoxon rank sum. We also computed their 99.0% bootstrap (10,000 rounds) confidence intervals. For the bootstrap test, we considered the null hypothesis *H0: KNN’s average accuracy is the same between different openness levels* and tested it against the alternative hypothesis *H1: KNN’s average accuracy significantly differs between different openness levels*. We reported the mean, standard deviation, and the 99.0% confidence interval for these tests.

For the Kruskal-Wallis and Friedman’s tests, we reported the effect size $r=\sqrt{\frac{{\chi }^{2}}{N}}$^35^ with *N* denoting the sample size and *χ*^2^ is the respective test-statistics. In the case of Wilcoxon tests, we used $r=\frac{W}{\sqrt{N}}$^36^ as effect size with *W* denoting the Wilcoxon statistics and *N* is the sample size. All results reported were Bonferroni corrected. All analyses were carried out in Python 2.7 and Matlab 2016a. We used Raincloud plots^37^ for visualization of the classification accuracies.

## Results

### Facial pre-touch clusters

**Figure 2:**
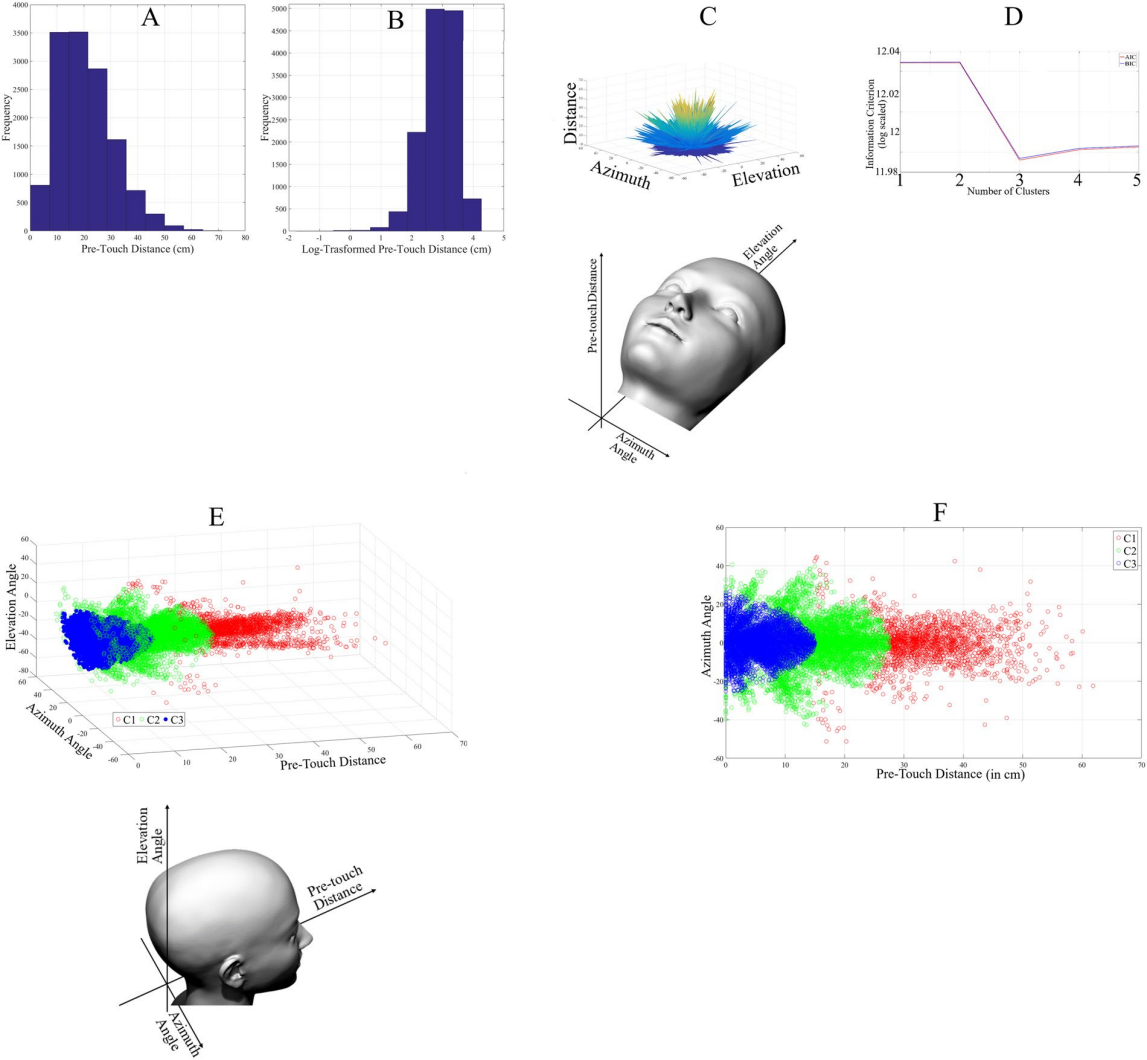
Facial pre-touch data of all the participants. (**A**) Distribution of actual facial pre-touch distances (in cm). (**B**) Distribution of log-transformed facial pre-touch distances. (**C**) 3D map of facial pre-touch distances in which the individuals’ preferential facial personal space are shown along the z-axis. The schematic diagram of the face direction is shown under this subplot. (**D**) Akaike (AIC in red) and Bayesian (BIC in blue) information criteria unanimously identify K = 3 as the best number of clusters for facial personal space. Their values are: AIC = [12.034, 12.034, 11.986, 11.991, 11.993] and BIC = [12.034, 12.034, 11.987, 11.991, 11.993]. (**E**) 3D facial pre-touch distance clusters: C1 (red), C2 (green), and C3 (blue). The schematic diagram of the face direction is shown under this subplot. (**F**) 2D facial pre-touch distance clusters that maps these distances against their corresponding azimuth angle.

We found that the actual (Fig. 2(A)) and log-transformed (Fig. 2(B)) facial pre-touch distances were not normally distributed (at 5.0% significance level; actual: p < 0.001, test-statistics = 0.07, M_*actual*_ = 20.55, SD_*actual*_ = 10.24, CI_*actual*_ = [20.40 20.69] and log-transformed: p < 0.001, test-statistics = 0.05, M_*log*−*transformed*_ = 2.88, SD_*log*−*transformed*_ = 0.57, CI_*log*−*transformed*_ = [2.88 2.89]). Figure 2(C) shows the 3D grids of the individuals’ facial personal space that is mapped along the azimuth and elevation angles associated with these distances around the face area. These angles were within (in degrees) azimuth ∈ [−51.30 44.47] and elevation ∈ [−63.84 48.30] intervals.

We applied K-means clustering on this grid to determine their grouping and used AIC and BIC (Fig. 2(D)) to identify the best number of clusters (k). Both these measures indicated that k = 3 (AIC_*k*=3_ = 11.986 and BIC_*k*=3_ = 11.987). In Fig. 2(D), values associated with *k* = 1,…, 5 are: AIC_*k*=1_ = 12.034, AIC_*k*=2_ = 12.034, AIC_*k*=3_ = 11.986, AIC_*k*=4_ = 11.991, AIC_*k*=5_ = 11.993 and BIC_*k*=1_ = 12.034, BIC_*k*=2_ = 12.034, BIC_*k*=3_ = 11.987, BIC_*k*_ = 11.991, BIC_*k*=5_ = 11.993. Figure 2(E) shows the resulting three clusters. We found that there were 1814, 5202, and 6440 facial pre-touch distance data points in C1 (M_*Distance*_ = 34.38, SD_*Distance*_ = 7.22, CI_*Distance*_ = [34.11 34.66], azimuth ∈ [−51.30 44.47], elevation ∈ [−63.84 48.30]), C2 (M_*Distance*_ = 15.87, SD_*Distance*_ = 6.01, CI_*Distance*_ = [15.73 16.01], azimuth ∈ [−42.78 40.52], elevation ∈ [−55.93 41.92]), and C3 (M_*Distance*_ = 5.89, SD_*Distance*_ = 3.95, CI_*Distance*_ = [5.81 5.97], azimuth ∈ [−25.32 26.73], elevation ∈ [−27.30 25.62]). These data points corresponded to twenty-seven, forty-seven, and forty-four participants. Figure 2(F) illustrates these clusters in 2D in which these distances are mapped against their corresponding azimuth angle.

### Facial pre-touch distance and openness correlation

**Figure 3:**
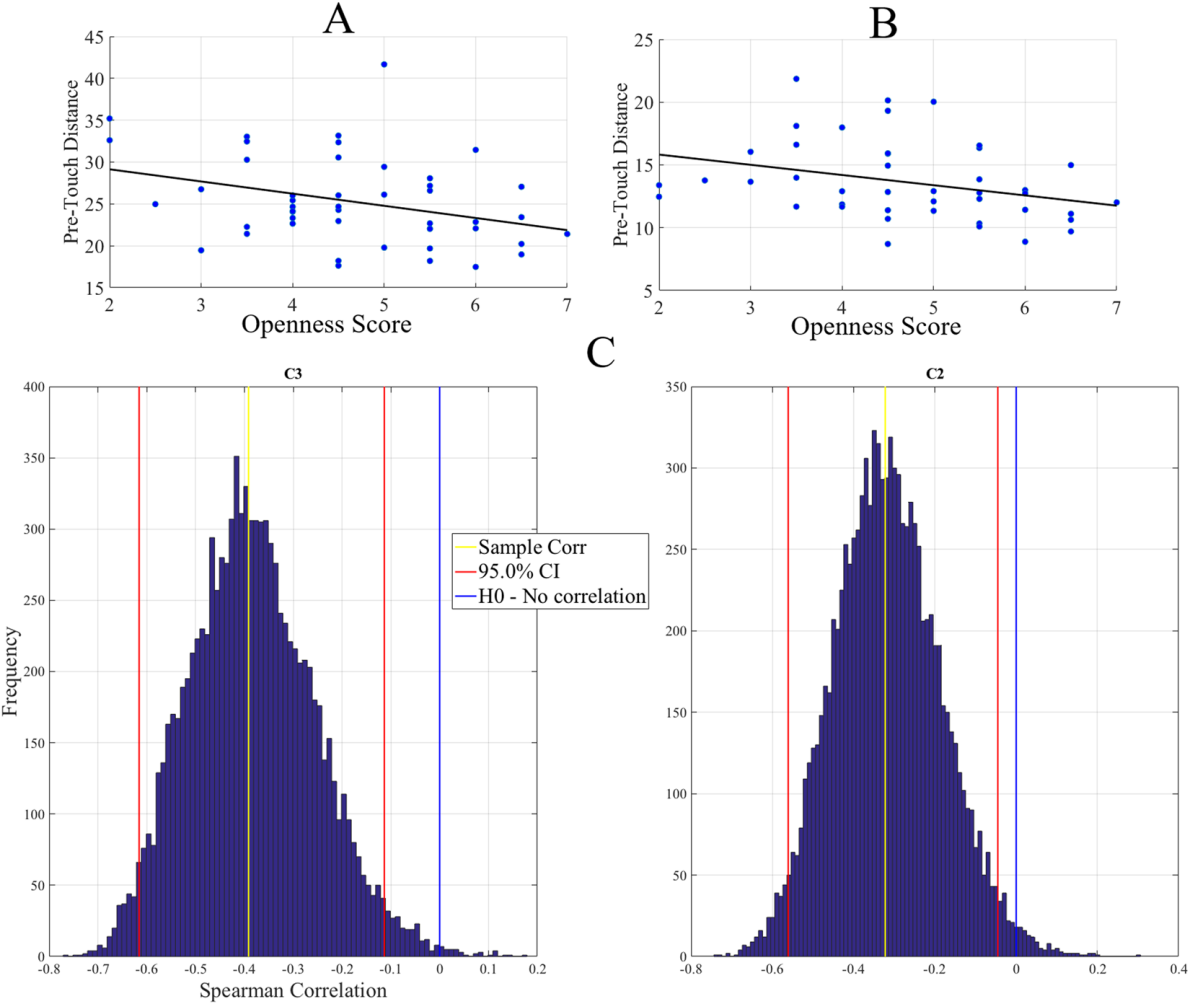
Openness (O) versus pre-touch distance Spearman correlations. (**A**) Cluster C2 (**B**) Cluster C3 (**C**) Bootstrap (10,000 simulation runs) 95.0% confidence intervals (CI) of the Spearman correlation between participants’ facial pre-touch distances and their FFM openness scores. The mean of the bootstrapped correlation coefficients is shown with the yellow line, the 95.0% confidence intervals are the two red lines, and the null hypothesis *H*0 (i.e., no correlation) is the blue line.

We found that the participants’ openness score showed a significant anti-correlation with their facial pre-touch distances in C2 (Fig. 3A) (r = −0.33, p < 0.03, M_*Distance*_ = 25.25, SD_*Distance*_ = 5.23, M_*O*_ = 4.67, SD_*O*_ = 1.20) and C3 (Fig. 3B) (r = −0.40, p < 0.01, M_*Distance*_ = 13.61, SD_*Distance*_ = 3.10, M_*O*_ = 4.72, SD_*O*_ = 1.23).

Table 1 summarizes the results of the bootstrap (Fig. 3C, 10,000 simulation runs) 95.0% confidence interval of these clusters’ correlation analysis. This table confirms the observed significant anti-correlation between the participants’ facial pre-touch distances and their FFM openness scores.

**Table 1:** Bootstrap (10,000 simulation runs) 95.0% confidence intervals (CI) associated with the correlation analysis of the facial pre-touch distance and the participants’ FFM openness scores.

Cluster	r	p-value (two-tailed)	CI_95.0%_
C2	−0.392	0.0084	[−0.621 −0.111]
C3	−0.322	0.0260	[−0.560 −0.046]

### Openness prediction

#### Overall prediction accuracy

**Figure 4:**
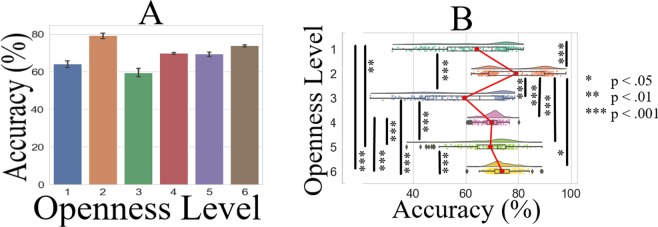
KNN accuracy. (**A**) Overall performance (i.e., six openness levels combined) and without considering the clusters. (**B**) Comparison of the accuracy between different openness levels and without considering the clusters. This figure illustrates the distribution of 200 simulation rounds in which we randomly assigned 30.0% of entire data to test set and used the remainder of data for training these models. While splitting the data, we also ensured that a proper proportion of each labels (i.e., 30.0% per label) was assigned to the test set. In this figure, the asterisks mark the significant differences between openness level prediction accuracies.

Kruskal-Wallis indicated (Fig. 4(A)) significant difference in KNN’s prediction accuracy on different openness level (p < 0.001, H(5, 1211) = 153.14, r = 0.36). Posthoc Wilcoxon tests (Fig. 4(B) and Table 2) revealed that KNN overall accuracy (i.e., C2 and C3 combined) in the case of openness level 1 was only higher than openness level 3 and below all the other openness levels. We also observed that KNN overall accuracy in the case of openness level 2 was higher than all the other openness levels. These results also indicated that whereas the overall accuracy in the case of openness level 4 was non-significant in comparison with the openness level 5, it was significantly lower than the overall accuracy in the case of level 6. We also observed that KNN overall accuracy in the case of openness level 6 was significantly higher than 5.

**Table 2:** Pair-wise Wilcoxon rank sum p-value, test-statistics, effect size, and the mean and standard deviation of the openness levels’ prediction accuracy by KNN (chance level accuracy ≈16.67%).

Paired Openness Levels	p<	W(60)	r	*M*_1_ (%)	*SD*_1_	*M*_2_ (%)	*SD*_2_
2	0.001	8.59	0.43	64.22	13.15	79.14	10.69
3	0.001	3.59	0.18	64.22	13.15	59.55	15.60
1 *versus*. 4	0.01	2.74	0.14	64.22	13.15	69.89	3.43
5	0.01	2.74	0.14	64.22	13.15	69.38	8.97
6	0.001	5.75	0.29	64.22	13.15	73.83	4.43
3	0.001	8.90	0.44	79.14	10.69	59.55	15.60
4	0.001	6.26	0.31	79.14	10.69	69.89	3.43
2 *versus*. 5	0.001	6.31	0.31	79.14	10.69	69.38	8.97
6	0.03	2.18	0.11	79.14	10.69	73.83	4.43
4	0.001	6.26	0.31	59.55	15.60	69.89	3.43
3 *versus*. 5	0.001	6.31	0.31	59.55	15.60	69.38	8.97
6	0.03	2.18	0.11	59.55	15.60	73.83	4.43
4 *versus*. 5	=0.38	0.88	0.04	69.89	3.43	69.38	8.97
6	0.03	2.18	0.11	69.89	3.43	73.83	4.43
5 *versus*. 6	0.001	6.31	0.31	69.89	3.43	73.83	4.43

The subscripts 1 and 2 in M and SD entries of this table refer to the first and the second items in each of the paired comparison.

**Figure 5:**
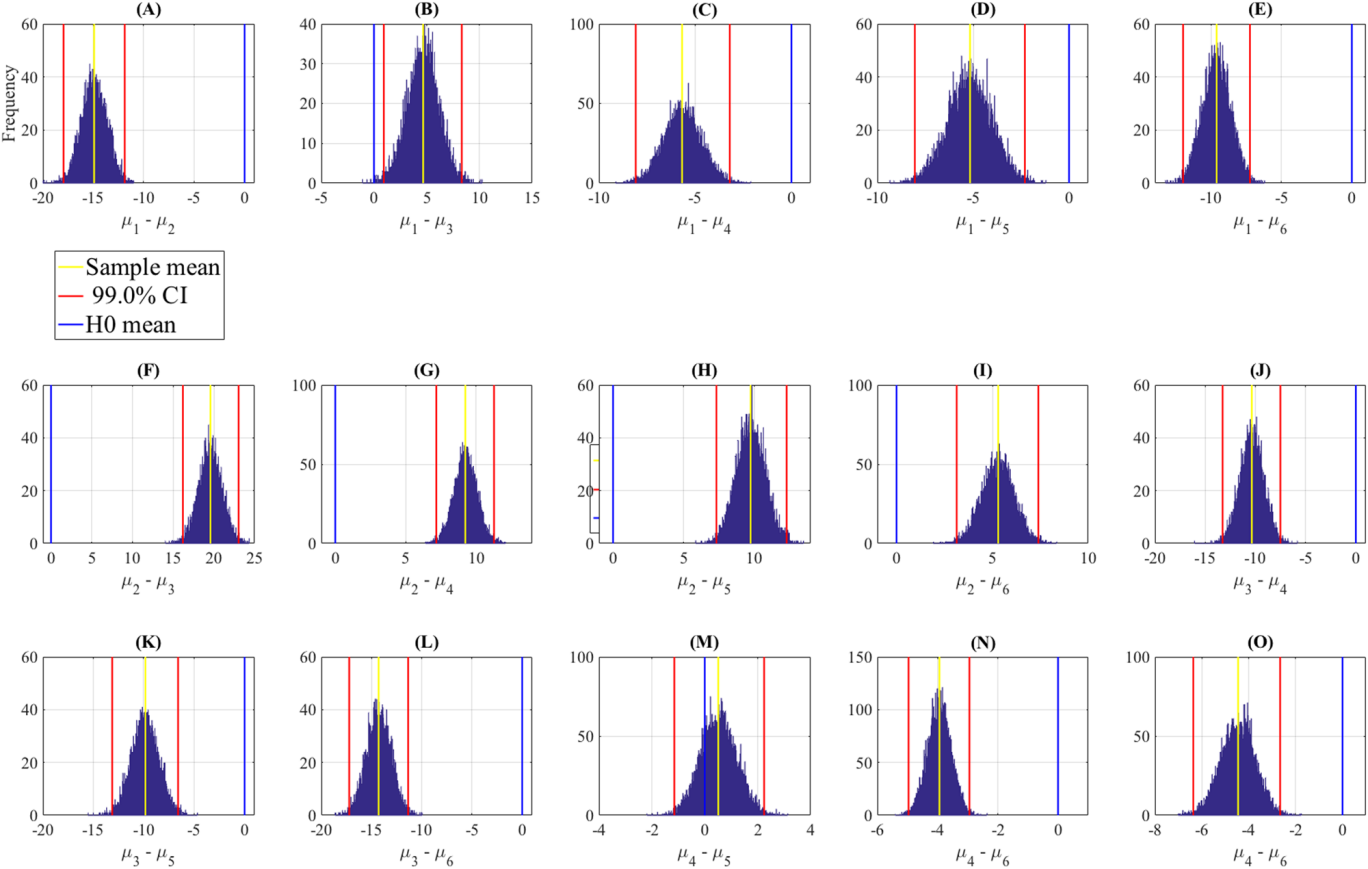
Bootstrap (10,000 simulation runs) 99.0% confidence intervals (CI) for comparative analysis of the overall (i.e., clusters C2 and C3 combined) KNN accuracy. These subplots correspond to the difference between openness levels (**A**) 1 vs. 2 (**B**) 1 vs. 3 (**C**) 1 vs. 4 (**D**) 1 vs. 5 (**E**) 1 vs. 6 (**F**) 2 vs. 3 (**G**) 2 vs. 4 (**H**) 2 vs. 5 (**I**) 2 vs. 6 (**J**) 3 vs. 4 (**K**) 3 vs. 5 (**L**) 3 vs. 6 (**M**) 4 vs. 5 (**N**) 4 vs. 6 (**O**) 5 vs. 6. For each paired comparison the sample mean difference (i.e., *μ*_*i*_−*μ*_*j*_, *i* = 1, …, 6, *j* = 1, …, 6) is shown with the yellow line, the 99.0% confidence intervals are the two red lines, and the null hypothesis *H*0 (i.e., mean difference is zero) is the blue line. Subplot (**M**) indicates that the comparative overall KNN performance (i.e., combined C2 and C3) between openness levels 4 and 5 is non-significant.

Figure 5 and Table 3 show the results of the bootstrap (10,000 simulation runs) confidence intervals for KNN’s overall accuracy (i.e., clusters C2 and C3 combined) paired openness levels. These results confirmed that KNN accuracy was significantly higher in the case of openness level 2 than all the other levels. They also indicated that its accuracy for the case of openness level 1 was only higher than openness level 3 (Fig. 5(B)) and lower than all the other labels. We also observed that whereas the accuracy for the openness level 4 showed no difference with respect to the level 5 (Fig. 5(M)) it was lower than that of the openness level 6 (Fig. 5(N)).

**Table 3:** Bootstrap (10,000 simulation runs) confidence intervals (CI) for comparative analysis of the overall (i.e., clusters C2 and C3 combined) KNN accuracy. comparative overall KNN performance (i.e., combined C2 and C3) between openness levels 4 and 5 is non-significant.

Paired Openness Levels	M<	SD	CI_99.0%_
2	−14.93	1.19	[−17.96 −11.89]
3	4.67	1.43	[0.92 8.32]
1 *versus*. 4	−5.66	0.95	[−8.08 −3.20]
5	−5.17	1.11	[−8.04 −2.30]
6	−9.61	0.96	[−12.01 −7.25]
3	19.59	1.33	[16.20 23.04]
4	9.26	0.79	[7.18 11.29]
2 *versus*. 5	9.77	0.98	[7.32 12.30]
6	5.29	0.82	[3.14 7.40]
4	−10.36	1.12	[−13.26 −7.50]
3 *versus*. 5	−9.86	1.27	[−13.12 −6.58]
6	−14.30	1.15	[−17.21 −11.34]
4 *versus*. 5	0.517	0.67	[−1.15 2.24]
6	−3.94	0.39	[−4.96 −2.94]
5 *versus*. 6	−4.45	0.72	[−6.36 −2.66]

The entries M and SD are the mean and the standard deviation of the calculated mean differences during the simulations.

#### C2 versus C3 predictions accuracy

**Figure 6:**
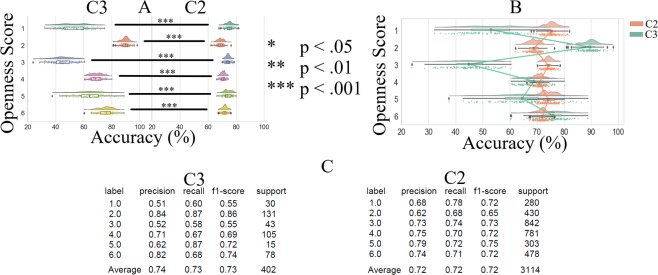
KNN accuracy. (**A**) C3 versus C2 in the case of within openness level. The asterisks mark the significant differences between openness level prediction accuracies. (**B**) Overlaid KNN accuracies for better visualization of the effect in clusters C3 and C2. (**C**) Precision, recall, and F1-score associated with KNN while predicting different openness level in C3 and C2. Column “Support” refers to the number of each openness levels that were included in each of these clusters’ test sets while testing the KNN predictions. The row “average” indicates the average precision, recall, and F1-score when all levels combined in their respective clusters.

Kruskal-Wallis indicated a significant difference between the accuracies in C2 and C3 (p < 0.001, H(1, 1211) = 64.30, r = 0.23). Posthoc tests identified (Fig. 6(A) and Table 4) that whereas KNN accuracy in the case of openness levels 2 and 6 were higher for the cluster C3 than cluster C2, it performed significantly better in C2 than C3 in the case of openness levels 1, 3, 4, and 5. Figure 6(B) shows the overlaid KNN accuracies for openness levels 1 through 6 in C2 and C3 for better visualization of the effect. Figure 6(C) shows the precision, recall, and F1-score associated with KNN while predicting different openness levels in C2 and C3. Column “Support” refers to the number of each openness levels that were included in each of these clusters’ test sets while testing the KNN predictions. The row “average” indicates the average precision, recall, and F1-score when all levels combined in their respective clusters.

**Table 4:** Level-wise prediction of the openness by KNN in C2 and C3: Wilcoxon rank sum p-value, test-statistics, effect size, and the mean and standard deviation of the openness levels’ prediction accuracy by KNN (chance level accuracy ≈ 16.67%).

Paired Openness Levels	p<	W(60)	r	*M*_*C*2_ (%)	*SD*_*C*2_	*M*_*C*3_ (%)	*SD*_*C*3_
1	0.001	12.14	0.85	75.45	2.72	52.98	9.23
2	0.001	12.28	0.86	68.81	2.27	89.46	3.02
3	0.001	12.28	0.86	74.24	1.66	44.86	7.10
4	0.001	3.62	0.25	70.88	1.66	68.90	4.34
5	0.001	8.03	0.57	74.24	2.82	64.52	10.30
6	0.001	6.56	0.46	71.87	1.98	75.80	5.26

**Figure 7:**
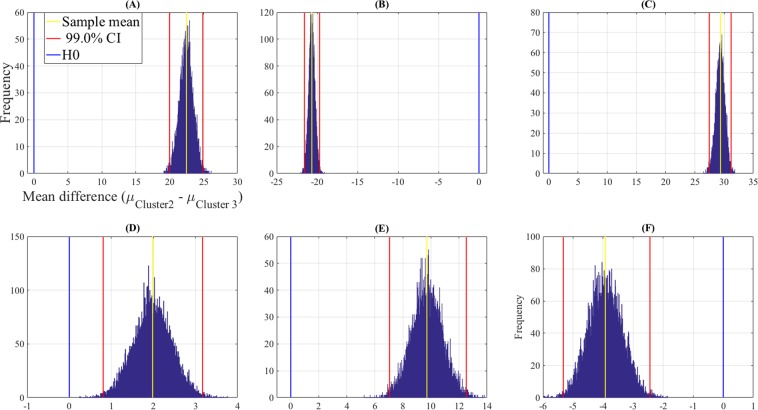
Bootstrap (10,000 simulation runs) 99.0% confidence intervals (CI) for KNN performance on clusters C2 and C3 for paired openness (**A**) level 1 (**B**) level 2 (**C**) level 3 (**D**) level 4 (**E**) level 5 (**F**) level 6. For each paired comparison the sample mean difference (i.e., *μ*_*C*2_−*μ*_*C*3_) is shown with the yellow line, the 99.0% confidence intervals are the two red lines, and the null hypothesis *H*0 (i.e., mean difference is zero) is the blue line. Whereas KNN performed significantly better in C3 for openness levels 2 (subplot (**B**)) and 6 (subplot (**F**)) its accuracy was significantly higher in C2 for openness levels 1 (subplot (**A**)), 3 (subplot (**C**)), 4 (subplot (**D**)), and 5 (subplot (**E**)).

Figure 7 and Table 5 show the results of the bootstrap (10,000 simulation runs) 99.0% confidence intervals for KNN performance on openness levels 1 through 6 in clusters C2 and C3. Entries of Table 5 confirm that while KNN achieved higher accuracies in C3 than C2 in the case of openness levels 2 (Fig. 7(B)) and 6 (Fig. 7(F)), its performance was significantly higher in C2 than C3 in the case of openness levels 1 (Fig. 7(A)), 3 (Fig. 7(C)), 4 (Fig. 7(D)), and 5 (Fig. 7(E)). However, we note that such a paired-wise difference was weaker in the case of openness level 4 (i.e., Fig. 7(D)) than the other five levels.

**Table 5:** Bootstrap (10,000 simulation runs) 99.0% confidence intervals (CI) for KNN performance on paired openness levels in C2 and C3. KNN accuracy was higher in C3 than C2 in the case of openness levels 2 and 3. On the other hand, it achieved significantly higher performance in C2 than C3 in the case of openness levels 1, 3, 4, and 5.

Paired Openness Levels	M	SD	CI_99.0%_
1	22.46	0.95	[19.98 24.87]
2	−20.66	0.37	[−21.57 −19.69]
3	29.37	0.72	[27.47 31.19]
4	1.98	0.46	[0.81 3.17]
5	9.73	1.06	[7.05 12.52]
6	−3.92	0.56	[−5.32 −2.44]

The entries M and SD are the mean and the standard deviation of the calculated mean differences during the simulations.

## Discussion

In this article we sought answer to the question of whether individuals’ personality traits are reflected in such tacit behavioural cues as preferred personal space. To examine this possibility, we considered a naturalistic scenario in which paired individuals signalled their preferred facial pre-touch distances. We considered the facial area touch interaction as opposed to other body parts that are more openly shared during social interactions (e.g., shoulder patting) due to higher sensitivity of people around their face which makes the facial boundary play a substantial role in understanding the people’s behavioural responses within the context of touch interaction.

The results of the cluster analysis of these facial pre-touch distances indicated potential patterns in individuals’ facial personal space in the form of three distinct subspaces. They also specified that within the first two immediate clusters around the face area these distance information significantly anti-correlated with individuals’ openness in FFM^8^. These results that were in line with the previous findings on peripersonal space representation^12^ and the effect of anxiety on such a space^14^ complemented the observations on the bodily maps of subjective feelings^25^ by providing evidence that such internal states are also present in our embodied interaction and its associated personal space^17–19^. They also extended the previous research that pointed at the connection between individuals’ personality and their brain functioning^1,5^ that can be traced throughout individuals’ development^38^ to the case of such immediate and observable behavioural responses as preferred personal space.

Our results also indicated that individuals’ sense of facial pre-touch space can significantly predict their personality trait of openness that was further categorized into six distinct groups. These results complemented the previous research that showed the personality traits^8^ can further be divided into four personality types^4^ by providing evidence on the correspondence between individuals’ preferred personal space and the level of openness in their personality in a finer-grain.

Although previous research pointed at the relation between individuals’ psychological characteristics and such behavioural responses as personal space^29^, these results suffered from lack of consensus on the interplay between personality and personal space^28^. Our results contributed to these previous findings by providing evidence that identified the role of individuals’ personality in shaping their personal space, thereby allowing for more informed conclusion on the influence of the personality traits on our behavioural responses and psychological capacities^2^. From a broader perspective, our results are potentially useful in such applied contexts as clinical settings related to psychopathological scenarios in which the patients’ acute psychological conditions directly affect their prospects about their inter/personal space and its boundary^39,40^.

Considering the fast emergence of embodied agents in our society^41,42^, our findings can also benefit the research in human-robot interaction (HRI) in which researchers urge for more robust evaluations that are founded on theoretical than sheer empirical approaches^43,44^ to enable these agents to better meet the grand social challenges^45^ [p.9] that these agents may encounter during their interaction with individuals^46^. For instance, an embodied agent that can determine the individuals’ level of openness using their preferred personal space during their interactions can better serve them when deployed for health-related interventions^47–49^.

There are several limitations in our study that require future consideration. Although our data included a moderately large number of samples, the small number of participants that only included younger adults does not allow for extension of our findings to all age groups (children, adolescents, and older people). In addition, our data did not include individuals from different geographical and cultural background. The absence of such a diversity that potentially plays a significant role in defining such trends as personal space and interpersonal distance does not allow for our results to be readily extended to all cultures and populations. Moreover, limiting the individuals’ behavioural responses to their facial area does not warrant the applicability of our results to overall embodied interaction of human subjects.

Our findings also highlight a challenge for future studies. Specifically, our results identified a significant correspondence between individuals’ openness and their personal space that predicted these individuals openness personality trait in six distinct categories. However, they left the utility of personal space and interpersonal distance in determination of other personality factors (e.g., neuroticism, agreeableness, etc.) unanswered. In this regard, we found a significant anti-correlation between individuals’ responses to questionnaires on openness and their degree of neuroticism (for details, see SM). Despite the possibility that such an observation might lead to expecting a relation between neuroticism and the personal space (e.g., the higher the neurotic feeling the larger the personal distance which opposes the results in the case of openness), we did not observe such a correspondence in our results. Future research can shed light on such potentially counterintuitive observations.

## Supplementary information

Supplementary Materials
