# Supplementary material for: Facial Pre-Touch Space Differentiates the Level of Openness Among Individuals
Source: Sci Rep. 2019 Aug 15;9:11924. doi: 10.1038/s41598-019-48481-x (PMC6695382; doi:10.1038/s41598-019-48481-x)
Supplement: Supplementary file 1 — Supplementary Materials [file 41598_2019_48481_MOESM1_ESM.pdf]

Facial Pre-Touch Space Differentiates the Level of Openness Among Individuals

Soheil Keshmiri[1,*], Masahiro Shiomi[1], Kodai Shatani[1,2], Takashi Minato[1], and Hiroshi Ishiguro[1,2]

[1]Advanced Telecommunications Research Institute International (ATR), Kyoto, Japan
[2]Graduate School of Engineering Science, Osaka University, Japan
[*]soheil@atr.jp

ABSTRACT

This Supplementary Material provides further results in support of our findings. Section 1 presents the results of the participants' FFM scores (i.e., extraversion, agreeableness, consciousness, openness, and neuroticism) Spearman correlation analysis. Section 2 presents the result of the Wilcoxon test on the familiarity effect. In Section 3 Kruskal-Wallis test to determine whether gender induced any effect on facial pre-touch distances is presented. Section 4 provides the correlation results between participants' facial pre-touch distances and their FFM scores in cluster C1. Results of these analyses in the case of clusters C2 and C3 with respect to FFM scores in extraversion, agreeableness, consciousness, and neuroticism are presented in Sections 5 and 6. In Section 7, we provide details analysis results on comparative analysis of the prediction accuracies between support vector classifier (SVC), quadratic discriminant analysis (QDA), adaboost, logistic regression (LR), naive Bayes (NB), random forest (RF), decision tree (DT), k-nearest-neighbour (KNN), and linear discriminant analysis (LDA). Our results indicate that KNN significantly outperform the other classifiers, thereby validating the choice of KNN in our main study.

1 FFM scores Correlation Analysis

We found (Fig. 1) that openness significantly correlated with extraversion (r = .43, $p < .01$). Similarly, we identified a significant correlation between agreeableness and conscientiousness (r = .34, $p < .03$). On the other hand, neuroticism showed significant anti–correlations with agreeableness (r = -.40, $p < .01$) and conscientiousness (r = -.40, $p < .01$).

2 Toucher-Evaluator Familiarity Effect

Wilcoxon signed rank (Figure 2) indicated non-significant familiarity effect between touchers and the evaluators based on the averaged first and last ten trials ($first10$ and $last10$ respectively) associated with each participant (p = .20, W(92) = 1.29, r = .13, $M_{first10}$ = 21.56, $SD_{first10}$ = 9.01, M_{last10} = 18.92, SD_{last10} = 8.87).

3 Gender Effect

Kruskal-Wallis indicated (Figure 3 (A)) no effect of gender on pre-touch facial distance among the paired participants (p = .38, H(3, 46) = 3.06, r = .26). Distribution of the participants' facial pre-touch distances based on their gender (i.e., male (1) and female (0)) as well as gender-group (i.e., MM (1), MF (2), FM (3), and FF (4)) are presented in Figure 3 (B) and (C). These plots are generated using tSNE[?] on participants' facial pre-touch distances.

4 C1 Spearman Correlation

We observed no correlation (Figure 4) between participants' facial pre-touch distances and their FFM scores (extraversion: r = -.10, p = .63, $M_{Distance}$ = 40.05, $SD_{Distance}$ = 8.99, M_E = 4.48, $SD_{Distance}$ = 1.30, agreeableness r = -.16, p = .42, M_A = 4.81, SD_A = 1.39, consciousness r = -.33, p = .10, M_C = 3.30, SD_C = 1.48, openness: r = -.13, p = .52, M_O= 4.56, SD_O = 1.23, neuroticism: r = .18, p = .37M_E = 4.33, SD_E = 1.59.

5 C2 Spearman Correlation

We found no correlation between participants' facial pre-touch distances and their FFM scores in extraversion (Figure 5 E) (r =.11, p = .44, $M_{Distance}$ = 25.25, $SD_{Distance}$ = 5.23, M_E = 4.31, SD_E = 1.41), agreeableness (Figure 5 A) (r = .12, p = .42, M_A = 4.78, SD_A = 1.23), consciousness (Figure 5 C) (r = -.15, p = .32, M_C = 2.99, SD_C = 1.38), and neuroticism (Figure 5 N) (r = .09, p = .54, M_N = 4.32, SD_N = 1.48).

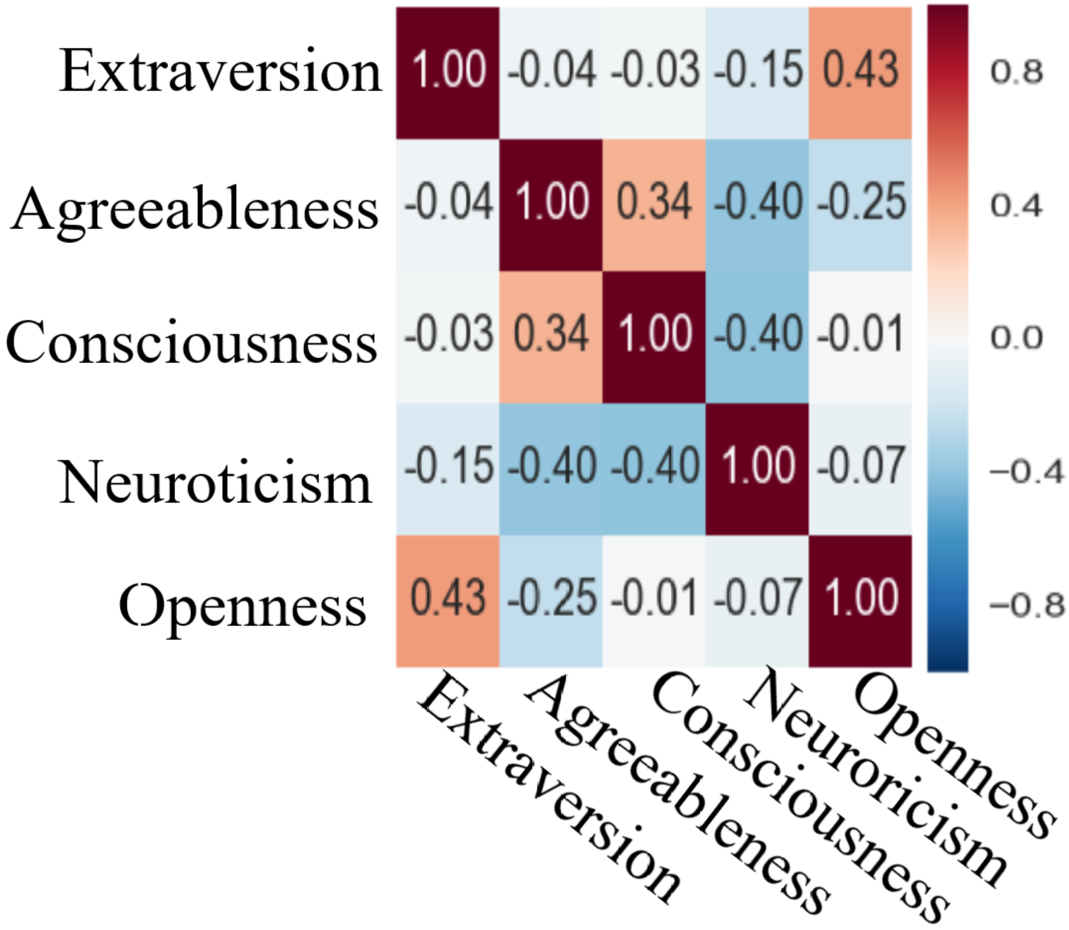

Figure 1. Spearman correlation between five factors: extraversion, agreeableness, consciousness, openness, and neuroticism.

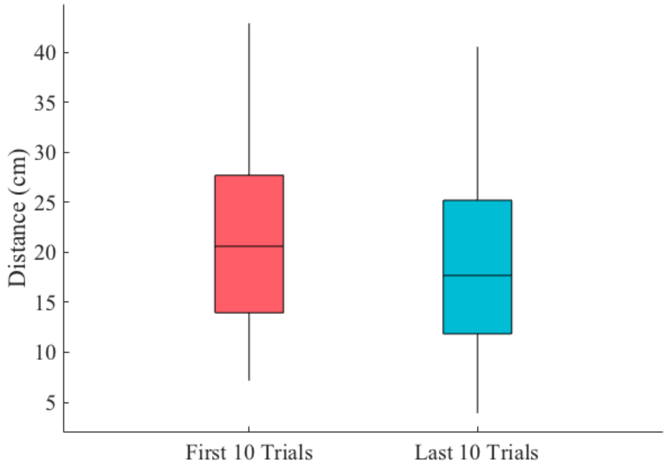

Figure 2. Wilcoxon two-sample test between the averaged first and last ten trials of the participants indicated no familiarity effect.

6 C3 Spearman Correlation

We found no correlation between participants' facial pre-touch distances and their FFM scores in extraversion (Figure 5 E) (r = .13, p = .41, $M_{Distance}$ = 13.61, $SD_{Distance}$ = 3.1, M_E = 4.34, SD_E = 1.42), agreeableness (Figure 5 A) (r = .14, p = .35, M_A = 4.78, SD_A = 1.17), consciousness (Figure 5 C) (r = -.06, p = .68, M_C = 3.03, SD_C = 1.36), and neuroticism (Figure 5 N) (r = .15,

p = .34, M_E = 4.41, SD_E = 1.46).

7 Models Overall Comparative Analysis

Friedman's test Friedman (Fig. 7 (A)) showed a significant difference between different models' accuracy ($p < .001$, H(8, 1799) = 1284.14, r = .84). Posthoc Wilcoxon indicated that KNN outperformed poorly in comparison with SVC ($p < .001$, W(398) = 17.30 r = .86, M_{KNN} = 72.28, SD_{KNN} = 1.33, M_{SVC} = 30.74, SD_{SVC} = 2.11), QDA ($p < .001$, W(398) = 17.30 r = .86, M_{QDA} = 42.25, SD_{QDA} = 9.16), adaboost ($p < .001$, W(398) = 17.30 r = .86, $M_{adaboost}$ = 36.20, $SD_{adaboost}$ = 5.83), LR ($p < .001$, W(398) = 17.30 r = .86, M_{LR} = 42.64, SD_{LR} = 8.66), NB ($p < .001$, W(398) = 17.30 r = .86, M_{NB} = 40.20, SD_{NB} = 7.19), RF ($p < .001$, W(398) = 17.30 r = .86, M_{RF} = 45.49, SD_{RF} = 11.11), DT ($p < .001$, W(398) = 17.30 r = .86, M_{DT} = 49.22, SD_{DT} = 12.39), and LDA ($p < .001$, W(398) = 17.30 r = .86, M_{LDA} = 41.32, SD_{LDA} = 8.77). This was followed by DT that significantly performed better than SVC ($p < .001$, W(398) = 17.30 r = .86), adaboost ($p < .001$, W(398) = 11.31, r = .57), LDA ($p < .001$, W(398) = 8.65, r = .43), RF($p < .001$, W(398) = 8.02, r = .40), QDA ($p < .001$, W(398) = 8.64, r = .43), NB ($p < .001$, W(398) = 8.64, r = .43), LR ($p < .001$, W(398) = 8.58, r = .43). We also observed that RF performed significantly better than SVC ($p < .001$, W(398) = 16.87, r = .84), LR ($p < .001$, W(398) = 5.10, r = .26), QDA ($p < .001$, W(398) = 7.05, r = .35), NB ($p < .001$, W(398) = 7.36, r = .37), adaboost ($p < .001$, W(398) = 9.01, r = .45), LDA ($p < .001$, W(398) = 7.82, r = .39). LR was also significantly better than SVR ($p < .001$, W(398) = 16.90, r = .85), NB ($p < .001$, W(398) = 6.12, r = .31), adaboost ($p < .001$, W(398) = 5.49, r = .27) while the difference between its performance remained non-significant with respect to LDA (p = .32, W(398) = .10, r = .05) and QDA (p = .87, W(398) = 2.54, r = .13). QDA was also better than SVC ($p < .001$, W(398) = 15.80, r = .79), NB ($p < .001$, W(398) = 3.59, r = .18), adaboost ($p < .001$, W(398) = 6.51, r = .33), LDA ($p < .001$, W(398) = 3.35, r = .17). Similarly, NB was able to outperform SVC ($p < .001$, W(398) = 15.72, r = .79), Adaboost ($p < .001$, W(398) = 11.65, r = .58). However, its performance difference remained non-significant with respect to LDA (p = .32, W(398) = .10, r = .05). Last, LDA significantly outperformed SVC ($p < .001$, W(398) = 14.21, r = .71) and adaboost ($p < .001$, W(398) = 14.21, r = .71). Figure 7 (B) shows these results. Figure 8 provides details on all models precision, recall, and F1-score associated with KNN

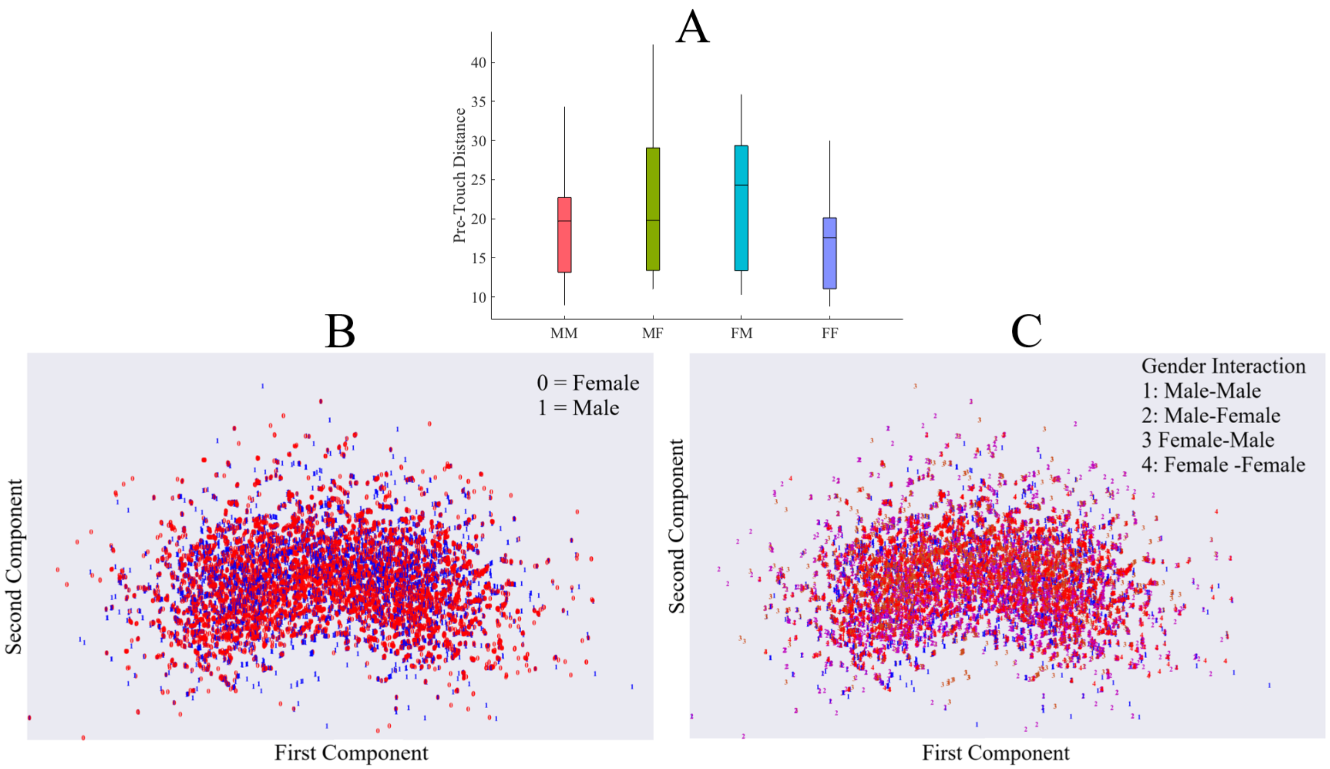

Figure 3. Gender effect: (A) Kruskall-Wallis test (male-male (MM), male-female (MF), female-male (FM), and female-female (FF)). (B) Distribution of the overall facial pre-touch distances among male (1) and female (0) participants. (C) Distribution of the overall facial pre-touch distances among MM (1), MF (2), FM (3), and FF (4) paired participants. Subplots (B) and (C) are based on application of t-distributed stochastic neighbour embedding (tSNE)[?] on participants' facial pre-touch distances.

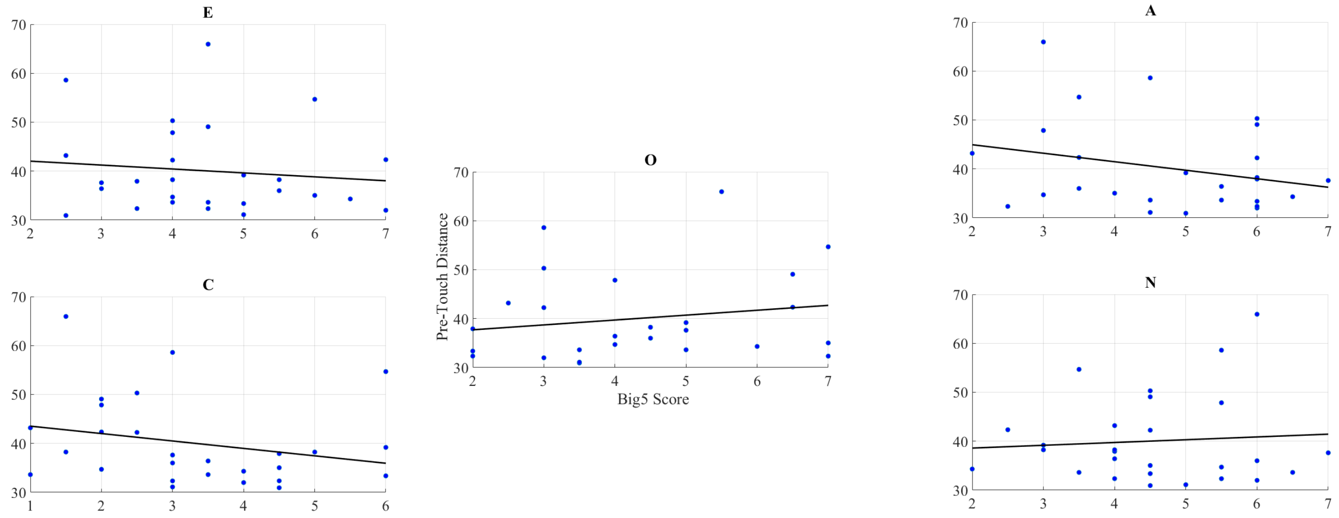

Figure 4. Cluster C1. Spearman correlation between extraversion (E), agreeableness (A), consciousness (C), openness (O), neuroticism (N), and the participants' pre-touch distances.

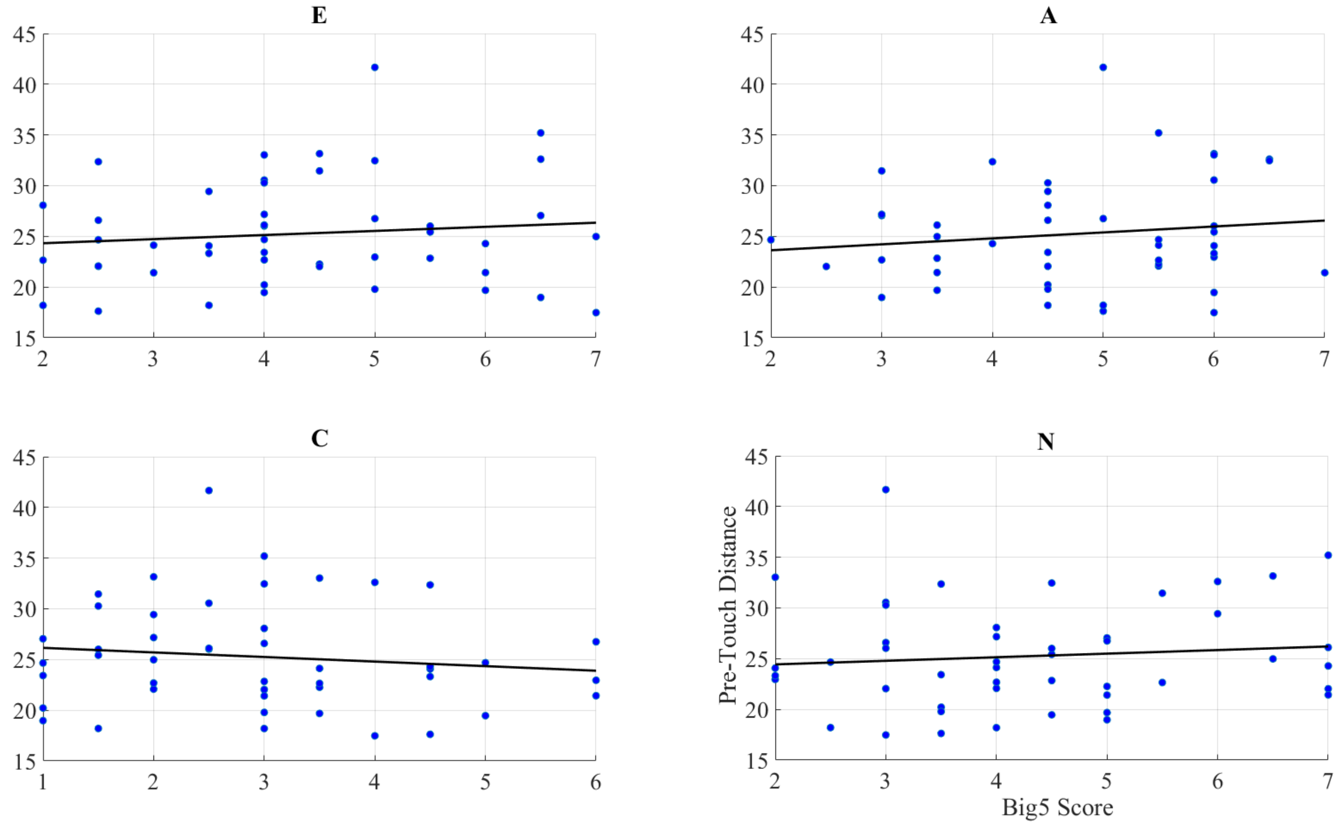

Figure 5. Cluster C2. Spearman correlation between extraversion (E), agreeableness (A), consciousness (C), neuroticism (N), and the participants' pre-touch distances.

while predicting different openness level in C3 and C2. Column "Support" refers to the number of each openness levels that were included in each of these clusters' test sets while testing the KNN predictions. The row "average" indicates the average precision, recall, and F1-score when all levels combined in their respective clusters.

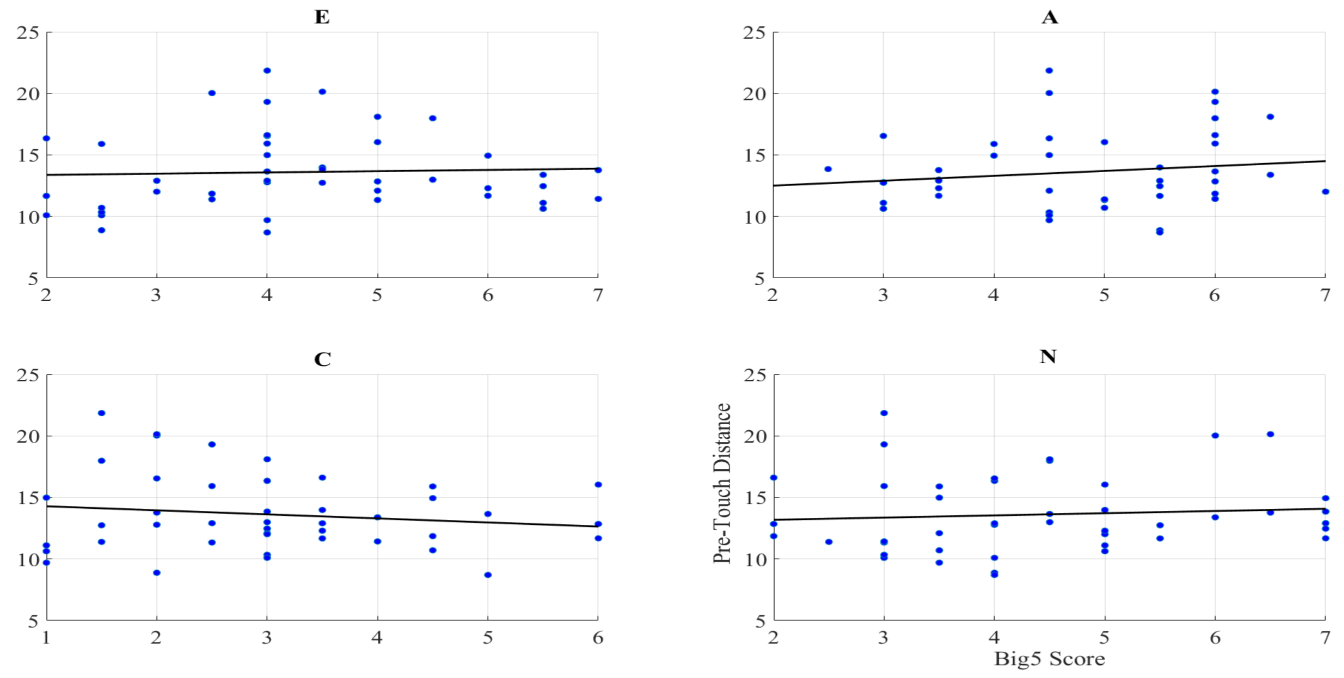

Figure 6. Cluster C3. Spearman correlation between extraversion (E), agreeableness (A), consciousness (C), neuroticism (N), and the participants' pre-touch distances.

8 Bootstrap Comparison: KNN versus the Other Models

Results of the 10,000 rounds of bootstrapping confirmed that the overall accuracy of KNN was significantly higher than SVC (Figure 9 (A), M = -41.56, SD = .18, CI = [-42.03 -41.12]), QDA (Figure 9 (B), M = -29.62, SD = .617, CI = [-31.21 -28.02]),

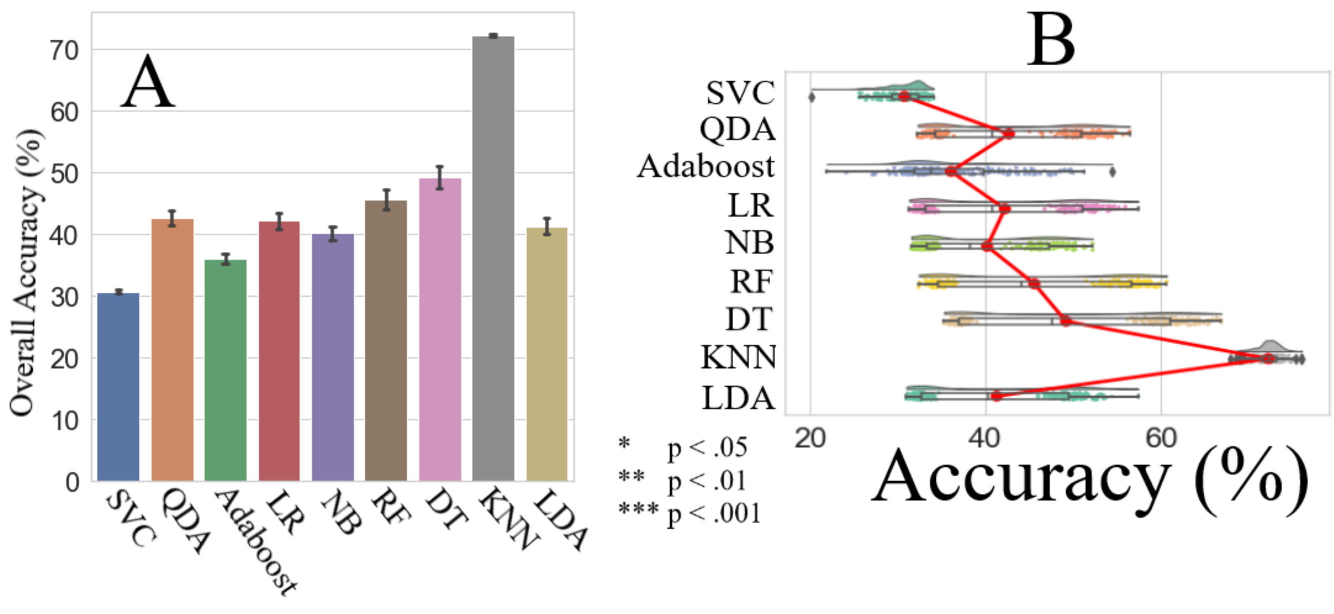

Figure 7. Model Accuracies Comparisons. (A) Models Overall prediction accuracies (B) Distribution of 200 simulation rounds, per model, in which we randomly assigned 30.0% of entire data to test set and used the remainder of data for training these models. We used the same train and test sets for all models. While splitting the data, we also ensured that a proper proportion of each labels (i.e., 30.0% per label) was assigned to the test set. In this figure, asterisks mark the significant difference between models' accuracy.

Adaboost (Figure 9 (C), M = -36.23, SD = .42, CI = [-37.31 -35.11]), LR (Figure 9 (D), M = -30.07, SD = .66, CI = [-31.77 -28.43]), NB (Figure 9 (E), M = -32.11, SD = .52, CI = [-33.44 -30.77]), RF (Figure 9 (F), M = -26.74, SD = .79, CI = [-28.78 -24.77]), DT (Figure 9 (G), M = -23.10, SD = .89, CI = [-25.34 -20.78]), and LDA (Figure 9 (H), M = -31.01, SD = .63, CI = [-32.66 -29.38]).

C3

	label	precision	recall	f1-score	support
KNN	1.0	0.51	0.60	0.55	30
	2.0	0.84	0.87	0.86	131
	3.0	0.52	0.58	0.55	43
	4.0	0.71	0.67	0.69	105
	5.0	0.62	0.87	0.72	15
	6.0	0.82	0.68	0.74	78
Average		0.74	0.73	0.73	402
DT	1.0	0.00	0.00	0.00	27
	2.0	0.76	0.86	0.80	141
	3.0	0.00	0.00	0.00	50
	4.0	0.54	0.78	0.64	95
	5.0	0.44	0.69	0.54	16
	6.0	0.56	0.62	0.58	73
Average		0.51	0.62	0.56	402
NB	1.0	0.27	0.33	0.30	42
	2.0	0.66	0.75	0.70	107
	3.0	0.00	0.00	0.00	57
	4.0	0.28	0.40	0.33	104
	5.0	0.38	0.14	0.21	21
	6.0	0.39	0.38	0.38	71
Average		0.36	0.41	0.38	402
LDA	1.0	0.00	0.00	0.00	39
	2.0	0.73	0.74	0.74	116
	3.0	0.00	0.00	0.00	48
	4.0	0.34	0.72	0.47	99
	5.0	0.00	0.00	0.00	18
	6.0	0.56	0.52	0.54	82
Average		0.41	0.50	0.44	402
SVC	1.0	0.00	0.00	0.00	34
	2.0	0.29	1.00	0.45	117
	3.0	0.00	0.00	0.00	58
	4.0	0.00	0.00	0.00	98
	5.0	0.00	0.00	0.00	27
	6.0	0.00	0.00	0.00	68
Average		0.08	0.29	0.13	402
RF	1.0	0.00	0.00	0.00	34
	2.0	0.56	0.93	0.70	113
	3.0	0.40	0.09	0.15	45
	4.0	0.46	0.68	0.55	94
	5.0	0.00	0.00	0.00	29
	6.0	0.65	0.48	0.55	87
Average		0.45	0.53	0.46	402
QDA	1.0	0.47	0.62	0.54	40
	2.0	0.74	0.76	0.75	123
	3.0	0.29	0.23	0.25	44
	4.0	0.43	0.54	0.48	94
	5.0	0.50	0.12	0.20	24
	6.0	0.57	0.48	0.52	77
Average		0.55	0.54	0.53	402
LR	1.0	0.80	0.12	0.21	33
	2.0	0.63	0.84	0.72	120
	3.0	0.25	0.02	0.04	41
	4.0	0.36	0.60	0.45	100
	5.0	0.00	0.00	0.00	22
	6.0	0.56	0.41	0.47	86
Average		0.49	0.50	0.45	402
Adaboost	1.0	0.27	0.70	0.39	37
	2.0	0.81	0.42	0.55	122
	3.0	0.36	0.12	0.18	41
	4.0	0.52	0.19	0.28	113
	5.0	0.08	0.32	0.13	22
	6.0	0.39	0.58	0.47	67
Average		0.52	0.37	0.39	402

C2

	label	precision	recall	f1-score	support
KNN	1.0	0.68	0.78	0.72	280
	2.0	0.62	0.68	0.65	430
	3.0	0.73	0.74	0.73	842
	4.0	0.75	0.70	0.72	781
	5.0	0.79	0.72	0.75	303
	6.0	0.74	0.71	0.72	478
Average		0.72	0.72	0.72	3114
DT	1.0	0.67	0.44	0.54	288
	2.0	0.41	0.08	0.13	433
	3.0	0.35	0.80	0.49	851
	4.0	0.91	0.05	0.10	727
	5.0	0.27	0.50	0.36	329
	6.0	0.37	0.20	0.26	486
Average		0.51	0.37	0.30	3114
NB	1.0	0.38	0.36	0.37	295
	2.0	0.01	0.00	0.00	430
	3.0	0.35	0.75	0.48	838
	4.0	0.35	0.40	0.38	758
	5.0	0.18	0.06	0.09	330
	6.0	0.00	0.00	0.00	463
Average		0.24	0.34	0.27	3114
LDA	1.0	0.37	0.22	0.27	281
	2.0	0.00	0.00	0.00	433
	3.0	0.38	0.78	0.51	852
	4.0	0.27	0.39	0.32	729
	5.0	0.06	0.02	0.03	339
	6.0	0.00	0.00	0.00	480
Average		0.21	0.33	0.24	3114
SVC	1.0	0.00	0.00	0.00	288
	2.0	0.00	0.00	0.00	437
	3.0	0.30	0.95	0.46	820
	4.0	0.34	0.24	0.28	787
	5.0	0.00	0.00	0.00	323
	6.0	0.00	0.00	0.00	459
Average		0.17	0.31	0.19	3114
RF	1.0	0.48	0.24	0.32	270
	2.0	0.00	0.00	0.00	427
	3.0	0.35	0.87	0.50	850
	4.0	0.30	0.33	0.32	769
	5.0	0.00	0.00	0.00	341
	6.0	0.00	0.00	0.00	457
Average		0.21	0.34	0.24	3114
QDA	1.0	0.33	0.30	0.32	277
	2.0	0.49	0.09	0.15	449
	3.0	0.37	0.82	0.51	865
	4.0	0.30	0.27	0.29	740
	5.0	0.35	0.12	0.18	321
	6.0	0.26	0.05	0.08	462
Average		0.35	0.35	0.29	3114
LR	1.0	0.40	0.09	0.14	285
	2.0	0.00	0.00	0.00	443
	3.0	0.37	0.77	0.50	841
	4.0	0.27	0.46	0.34	738
	5.0	0.00	0.00	0.00	323
	6.0	0.00	0.00	0.00	484
Average		0.20	0.33	0.23	3114
Adaboost	1.0	0.24	0.43	0.31	271
	2.0	0.41	0.16	0.23	446
	3.0	0.44	0.41	0.43	893
	4.0	0.27	0.22	0.24	719
	5.0	0.31	0.55	0.40	328
	6.0	0.21	0.21	0.21	457
Average		0.33	0.32	0.31	3114

Figure 8. All models precision, recall, and F1-score associated with KNN while predicitng different openness level in C3 and C2. Column ”Support” refers to the number of each openness levels that were included in each of these clusters’ test sets while testing the KNN predictions. The row ”average” indicates the average precision, recall, and F1-score when all levels combined in their respective clusters.

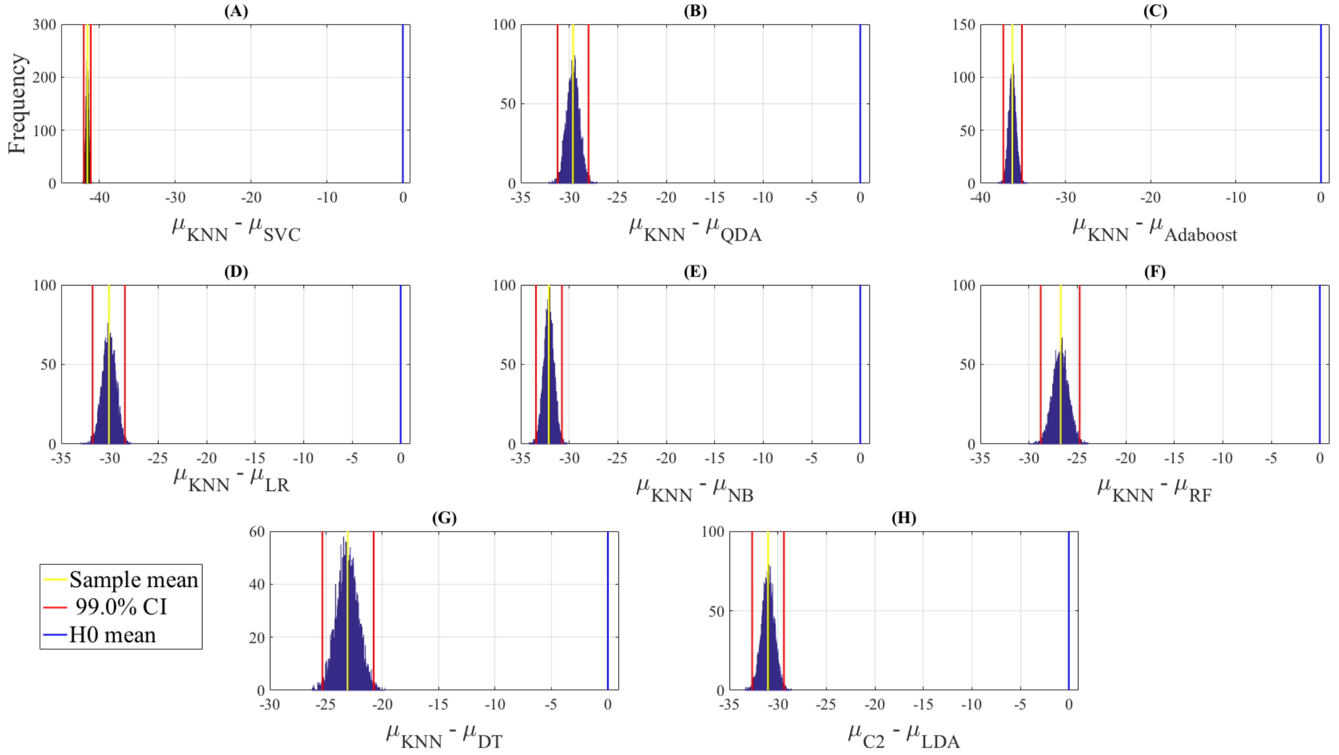

Figure 9. Bootstrap 99.0% confidence intervals for overall KNN accuracy versus (A)support vector classifier (SVC) (B) quadratic discriminant analysis (QDA) (C) Adaboost (D) logistic regression (LR) (E) naive Bayes (NB) (F) random forest (RF) (G) decision tree (DT) (H) linear discriminant analysis (LDA). Large margin of significantly higher accuracy achieved by KNN in comparison to these other classifiers is evident in this subplots. In this subplots $H0$ refers to the null hypothesis that the difference between the averaged accuracies of the two compared models is zero (i.e., $H0 : \mu KNN - \mu mdl = 0$ where mdl refers to either of the other models in subplots (A) through (H)).
